# Sleep, Physical Activity, and Mood Among People Seeking Mental Health Care

**DOI:** 10.1001/jamanetworkopen.2026.1194

**Published:** 2026-03-09

**Authors:** Aishani Kulshreshtha, Yu Fang, Elizabeth D. Mills, Amy S. B. Bohnert, Srijan Sen

**Affiliations:** 1 Gilbert S. Omenn Department of Computational Medicine and Bioinformatics, University of Michigan, Ann Arbor; 2 Michigan Neuroscience Institute, University of Michigan, Ann Arbor; 3 Department of Anesthesiology, Michigan Medicine, Ann Arbor; 4 Eisenberg Family Depression Center, University of Michigan, Ann Arbor

## Abstract

**Importance:**

Improving mental health through targeting behaviors like sleep and physical activity in treatment has been challenging, in part due to the challenge of measuring these factors in an accurate manner. Mobile technology can enable an understanding of the dynamic, complex associations between physical activity and sleep, but most prior mobile technology studies have had modest sample sizes and utilized cross-sectional, between-person designs, thus limiting their impact.

**Objective:**

To investigate the within-person associations between sleep, physical activity, and daily mood among individuals receiving mental health treatment to inform health behavior recommendations.

**Design, Setting, and Participants:**

This 12-month cohort study involved patients seeking mental health care at the University of Michigan academic medical center mental health care clinics. All participants were enrolled between May 13, 2020, and December 12, 2022. Data analysis was performed from September 2024 to June 2025.

**Exposures:**

Objective, wrist-based actigraphy measures of sleep (total sleep time, interrupted nighttime sleep, and napping) and physical activity (step count) were obtained.

**Main Outcomes and Measures:**

The primary outcome was participant-reported daily mood score, on a scale of 1 (worst mood) to 10 (best mood). Linear mixed-effects models were used to estimate associations among sleep, physical activity, and mood scores.

**Results:**

A total of 1476 participants (mean [SD] age, 36.5 [14.2] years; 1062 [72.0%] female) were included in the analysis. Sleep duration was associated with subsequent mood through an inverse U-shaped relationship, with both short and long sleep duration associated with poorer mood (quadratic term *b* = 0.027; 95% CI, −0.031 to −0.023). Notably, patients varied substantially in their optimal sleep duration (mean [SD], 6.8 [1.9] hours) for peak mood. Physical activity was positively associated with subsequent mood (linear term *b* = 0.160; 95% CI, 0.149 to 0.162; *P* < .001; quadratic term *b* =− 0.022; 95% CI, −0.027 to −0.017; *P* < .001), with diminishing associations at higher than usual activity for that individual. Conversely, daily mood was associated with subsequent sleep (linear term *b* = −1.377; 95% CI, −1.877 to −0.877; *P* < .001; quadratic term *b* = −0.394; 95% CI, −0.765 to −0.023; *P* = .037) and step count (linear term *b* = 0.020; 95% CI, 0.003 to 0.030; *P* = .02; quadratic term *b* = −0.010; 95% CI, −0.020 to −0.001; *P* = .03) in a wavelike manner.

**Conclusions and Relevance:**

This cohort study of people seeking mental health care found complex, bidirectional associations between sleep, physical activity, and mood with individual variation in optimal sleep duration for mood scores. The findings advance progress toward effectively targeting health behaviors to improve mental health.

## Key Points

**Question:**

What is the association of sleep and physical activity with mood score in patients seeking mental health care?

**Findings:**

This cohort study of 1476 patients seeking mental health care revealed dynamic, reciprocal associations between mood, physical activity, and sleep, as documented through actigraphy and daily mood diaries. Patients varied substantially in their optimal sleep duration for mood, with both shorter and longer sleep than a person’s optimal duration being significantly associated with worse subsequent mood.

**Meaning:**

These findings suggest that knowledge of individual sleep-mood and activity-mood patterns may help to develop more focused and personalized behavioral strategies to improve mental health and mood.

## Teaser

This cohort study investigates the associations between sleep, physical activity, and daily mood score in patients seeking mental health care.

## Introduction

Mental health conditions affect approximately one-half of the global population at some point in their lifetime and are the leading cause of years lived with a disability.^1^ Sleep and physical activity are 2 key modifiable factors that have been linked to mental health.^2,3^ However, little progress in improving mental health through targeting these modifiable factors has been made, in part because fully understanding the complex, granular associations between these factors has been challenging.

While studies to date suggest an association between sleep duration and mood, no clear consensus on the nature of the association has emerged, with studies identifying linear,^4,5^ inverse U-shaped,^6,7,8,9,10,11^ inverse J-shaped,^12,13^ arbitrarily nonlinear,^14^ or even null^15^ associations. In parallel, while studies have consistently found a positive association between physical activity and mood,^16^ studies conflict on whether there is a unidirectional association^17^ or bidirectional^15,18^ association and on the shape (eg, whether it is linear) of the association between activity and mood.

Traditionally, studies have relied on retrospective self-reported measures, which are prone to recall and social desirability biases.^6,7,8,9,12,13,19,20^ The growing use of mobile technology has enabled collection of objective, real-time sleep, and physical activity data. However, design limitations have constrained the impact of mobile technology work conducted to date. Specifically, studies have been limited by their small sample sizes, which affects power to understand the shape of associations, individual differences, and generalizability of findings.^3,5,21,22^ Furthermore, studies have predominantly used cross-sectional designs and primarily focused on between-person associations, choices that limit the assessment of casual and bidirectional associations between sleep and physical activity with mood.^5,6,9,13,14,23,24^ Finally, many mobile technology studies are limited by their short study time frames, usually between 7 and 14 days.^15,25,26,27^ Such brief time periods may be insufficient to capture the complex temporal dynamics and long-term interrelationships of sleep, physical activity, and mental health. Longer follow-up periods are crucial for studies with within-person designs; they help improve estimation and reliability of findings by providing sufficient repeated measures per participant. To address these limitations, the present study asks whether and how within-person, linear, and nonlinear deviations in sleep and physical activity are bidirectionally associated with daily mood among patients seeking psychiatric services across a 12-month time period.

## Methods

### Study Design and Participants

This cohort study included 2079 adult outpatients seeking mental health care enrolled between May 13, 2020, and December 12, 2022, who were followed up for 12 months. Potential participants were those with a forthcoming mental health appointment through the University of Michigan health care system between 2020 and 2022. Written informed consent was obtained from all participants. The University of Michigan institutional review board approved all procedures in the study. Additional details of the recruitment and protocols are available in Horwitz et al^28^ and Saulnier et al.^29^ This study followed the Strengthening the Reporting of Observational Studies in Epidemiology (STROBE) reporting guideline for cohort studies.

### Assessments

We assessed age, sex, race, educational level, employment status, 9-item Patient Health Questionnaire (PHQ-9) score (range, 0-21, with higher scores indicating higher depression symptoms),^30^ and 7-item Generalized Anxiety Disorder (GAD-7) score (range, 0-21, with higher scores indicating higher anxiety symptoms),^31^ along with other psychological and historical information through a baseline survey that participants completed at the start of the study. Race was self-reported by participants during their baseline survey as American Indian or Alaska Native, Asian, Black or African American, multiple races, White, other, or prefer not to answer. Other was used to self-identify as belonging to an additional group not explicitly listed. Data on race are included in this study for demographic characterization. Through a smartphone application (MyDataHelps; CareEvolution), participants completed daily mood ratings in response to a notification at 6:00 pm with the prompt, “On a scale of 1 (worst mood) to 10 (best mood), what was your average mood today?”

Wearable devices (Fitbit Charge 3, Inspire HR, or Inspire 2 [Google, Inc]) were used to collect sleep and physical activity data. As newer versions were introduced, these models were adopted sequentially over the study period. All 3 device models use the same underlying sensor technologies, supporting comparability.^32^

### Data Processing

For sleep measures, 24-hour total sleep time (TST) for a particular day was defined as the sum of durations of all sleep episodes with wake time between 12:00 am and 11:59 pm that day. On days with multiple sleep episodes, the longest sleep episode was categorized as the primary sleep episode. Consistent with prior literature,^33,34^ we excluded step counts less than 1000 or more than 30 000 per day (eFigure 1A in Supplement 1) and sleep episodes with durations shorter than 10 minutes or longer than 16 hours (eFigure 1B in Supplement 1).

Steps and sleep duration were standardized for each participant according to their own personal average and variability to assess associations at the within-person level. Person-days with standardized values greater than 3 SD above or below the personal mean were removed. On days with multiple sleep episodes, if the gap between episodes was less than 5 minutes, the episodes were merged and treated as a single episode. Then, if the additional episode occurred within less than 3 hours of the primary sleep episode, it was classified as an interrupted sleep episode; otherwise, it was defined as a nap.

Participants who had no change in their mood ratings during the course of the study were eliminated from the mood data as they did not present any within-person variability. Finally, we excluded participants who had missing demographic data and fewer than 7 days of data.

### Statistical Analysis

Data analysis was performed from September 2024 to June 2025. We sought to assess the within-person associations between mood, physical activity, and sleep. The daily estimated variables (TST, steps, and mood) were all person-mean centered. We estimated 4 linear mixed-effects models: (1) the association of TST with subsequent mood, (2) the association of daily mood with subsequent TST, (3) the association of daily step count with subsequent mood, and (4) the association of mood with subsequent step count. We first fitted models with just linear terms for variables of interest, then added quadratic terms to compare the goodness-of-fit between the 2 models using Akaike information criterion (AIC). To capture individual baseline differences in outcome levels, each model included a random intercept for every participant. Models were adjusted for weekend or weekday, day of the study, as well as age, sex, race, educational level, and employment status. Additionally, we included lagged variables (ie, previous-day values of mood, steps, or sleep) corresponding to the outcome variable in each model to account for temporal dependencies and to account for prior states. Finally, to assess the association of different types of multiple sleep episodes throughout the day with mood, we classified sleep each day into 4 categories (only a single episode, primary plus nap, primary plus interrupted sleep, and primary plus nap plus interrupted sleep) and included this variable in model 1.

To determine the optimal range of sleep durations associated with peak mood for each participant with at least 7 days of data, we used the estimated inverse U-shaped curve obtained from the model for association of sleep with subsequent mood. Since TST was standardized for each participant in the model, the mood estimates could be personalized to each participant’s own mean and SD of sleep, allowing us to calculate their optimal sleep duration. For each participant, we generated estimated mood values on the basis of the estimated curve across their range of sleep durations while maintaining the observed levels for all other covariates. The sleep minutes corresponding to the maximum estimated mood was defined as the participant’s optimal sleep duration.

Additional sensitivity analyses using different thresholds to define nap vs interrupted sleep were conducted to test robustness of results (see the eMethods in Supplement 1). Furthermore, to assess robustness across symptom severity, we re-estimated the models stratified by baseline PHQ-9 (depression, cutoff ≥10)^35^ and GAD-7 (anxiety, cutoff ≥10)^31^ scores. We also stratified the sleep-to-mood model by sleep pattern groups (consistently long vs consistently short vs inconsistent vs typical sleepers; see the eMethods Supplement 1 for definitions) to compare the magnitude of sleep-mood associations across these groups. Finally, we estimated a combined model including both sleep and steps as simultaneous factors of mood to assess whether their associations were independent.

All analyses were conducted in R statistical software version 4.4.1 (R Project for Statistical Computing)^36^ using the lmerTest package.^37^ The *P *values reported were 2-sided, with significance defined as *P* < .05.

## Results

### Sample Characteristics

A total of 1476 participants provided sufficient mood score, sleep duration, and physical activity data for inclusion (eFigure 2 in Supplement 1). The sample had a mean (SD) age of 36.5 (14.2) years (age range, 18-79 years), and most participants were female (1062 participants [72.0%]) who held a bachelor’s degree or higher (864 participants [58.6%]) (Table 1). These participants provided an average of 120 days with mood, sleep, and physical activity data all present, consisting of 178 403 daily observations throughout the study period. Participants reported a mean (SD) mood score of 6.3 (1.8). Mean (SD) step counts recorded were 6660 (4260) steps, and the mean (SD) TST was 6.98 (1.95) hours.

**Table 1.** Sample Characteristics

Characteristics	Participants, No. (%) (N = 1476)
Age, mean (SD), y	38.4 (14.3)
Sex	
Female	1062 (72.0)
Male	404 (27.4)
Prefer not to answer	10 (0.7)
Employment status	
Full-time	576 (39.0)
Part-time	277 (18.8)
Retired	94 (6.4)
Student	205 (13.9)
Unemployed	324 (22.0)
Race^a^	
American Indian or Alaska Native	4 (0.3)
Asian	85 (5.8)
Black or African American	105 (7.1)
Multiple races	75 (5.1)
Prefer not to answer	16 (1.1)
White	1145 (77.6)
Other^b^	46 (3.1)
Education	
Associate degree	135 (9.2)
Bachelor’s degree	403 (27.3)
Doctorate degree	80 (5.4)
High school graduate or General Educational Development test	161 (10.9)
Less than a high school diploma	31 (2.1)
Master’s degree	246 (16.7)
Some college credit	359 (24.3)
Trade, technical, or vocational training	61 (4.1)

^a^ Race was self-identified.

^b^ Other race was self-identified as something else.

### Daily Mood and Sleep Duration

**Figure 1.**
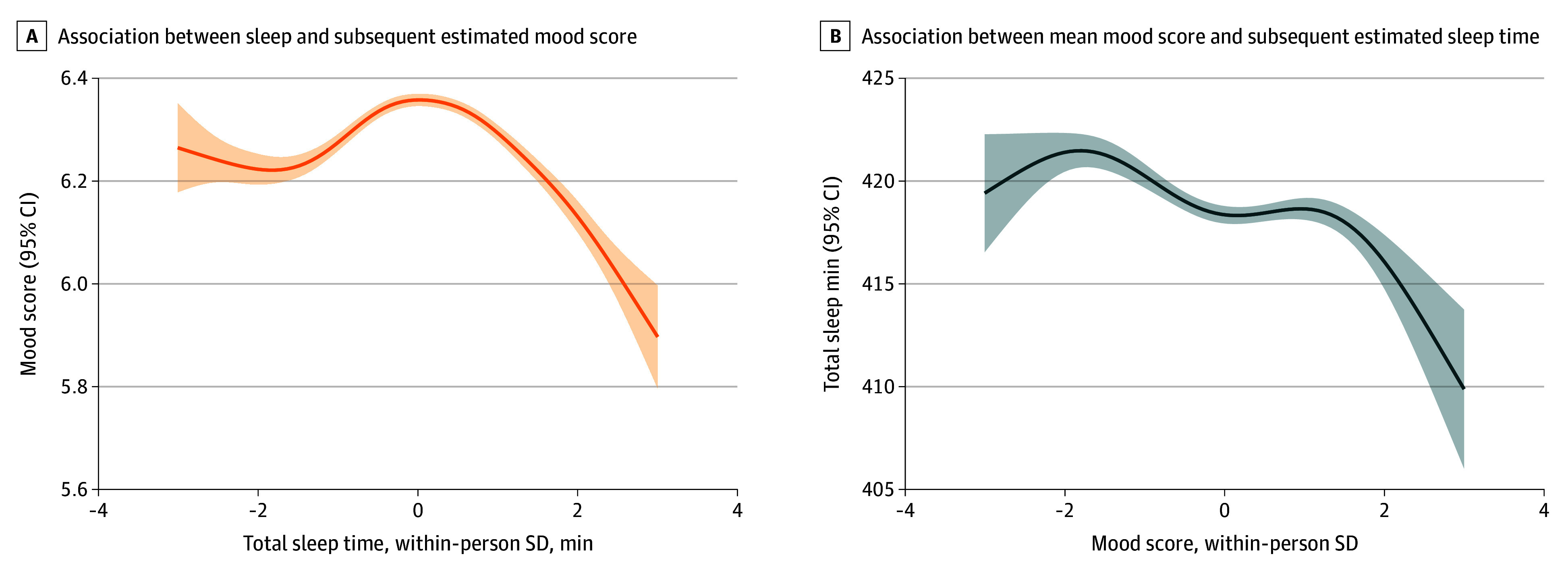
Line Graphs of Associations Between Total Sleep Duration and Mood Score Graphs show model-estimated outcome values using generalized additive model smoothing for visualization of within-person associations. A, Within-person association of total sleep time (SD from person mean; observed range, −3 to 3 SD) with estimated subsequent mood score (range, 1-10). B, Within-person association of mood (SD from person mean; observed range, −3 to 3 SD) with subsequent night’s estimated total sleep time (in minutes). Shaded regions represent 95% CIs.

First, we estimated the within-person association between TST and subsequent mood. The quadratic model provided substantially better fit than the linear model (difference in AIC = −142.37) The model revealed an inverse U-shaped trend (Table 2 and Figure 1A). Specifically, at shorter sleep durations, mood improved as sleep duration increases, but beyond 1 SD from participants’ average sleep, longer sleep durations were associated with lower mood (quadratic term *b* = −0.027; 95% CI, −0.031 to −0.023; *P* < .001). The inverse U-shaped sleep-mood association remained robust when stratified by sleep pattern groups (eTable 1 in Supplement 1). The mean (SD) TST associated with the maximum estimated mood was 6.8 (1.9) hours, and on average the optimal sleep window ranged from 6.2 to 7.3 hours between participants. Across the full sample, a within-person 1 SD deviation from optimal sleep duration was associated with −0.027-point mood linear change. For 297 participants (20.1%), the estimated optimal TST fell outside 1 SD above or below their own mean TST. Additionally, there was a significant negative association between the presence of additional nonprimary sleep episode types (interrupted sleep, *b* = −0.130; 95% CI, −0.155 to −0.105; *P* < .001; nap, *b* = −0.180; 95% CI, −0.198 to −0.161; *P* < .001; both, *b* = −0.269; 95% CI, −0.319 to −0.218; *P* < .001) and subsequent mood, indicating that, given the same TST, having multiple sleep episodes (regardless of type) is associated with worse subsequent mood, compared with having a single primary sleep episode. Sensitivity analyses with alternate thresholds for defining naps revealed that findings remained consistent (eTable 2 in Supplement 1).

**Table 2.** Bidirectional Associations Between Total Sleep Time and Subsequent Mood Score

Model and variable	*b* (95% CI) [SE]	*P* value
Sleep-to-mood model^a^		
Total sleep time, scaled (linear term)	−0.002 (−0.008 to 0.005) [0.003]	.58
Total sleep time, scaled (quadratic term)	−0.027 (−0.031 to −0.023) [0.002]	<.001
Sleep episode type (non-nap)	−0.130 (−0.155 to −0.105) [0.013]	<.001
Sleep episode type (nap)	−0.180 (−0.198 to −0.161) [0.009]	<.001
Sleep episode type (both)	−0.269 (−0.319 to −0.218) [0.026]	<.001
Mood-to–total sleep time model^b^		
Mood, scaled (linear term)	−1.377 (−1.877 to −0.877) [0.255]	<.001
Mood, scaled (quadratic term)	−0.394 (−0.765 to −0.023) [0.189]	.04

^a^ The sleep-to-mood model was adjusted for age, sex, employment status, educational level, race, day in study, day in week, and previous day’s mood (conditional *R*^2^ = 0.451; marginal *R*^2^ = 0.179).

^b^ The mood-to–total sleep time model was adjusted for age, sex, employment status, educational level, race, day in study, day in week, and previous day’s total sleep time (conditional *R*^2^ = 0.288; marginal *R*^2^ = 0.034).

Next, we estimated the association of within-person mood with TST the following day, while adjusting for covariates and previous night’s TST (Table 2). Quadratic model provided a better fit than linear model (difference in AIC = −2.33). There was a significant negative association between the linear (*b* = −1.377; 95% CI, −1.877 to −0.877; *P* < .001) and quadratic (*b* = −0.394; 95% CI, −0.765 to −0.023; *P* = .037) terms for within-person variation in mood and TST the following day. The participants’ TST tended to decrease when their mood deviated either higher or lower than their usual levels. Estimated TST increased slightly as mood deviated up to roughly 2 SD above or below a participant’s usual level and then declined beyond those extremes (Figure 1B). Overall, both models highlight a significant bidirectional and nonlinear association between TST and mood.

### Daily Mood and Activity

**Figure 2.**
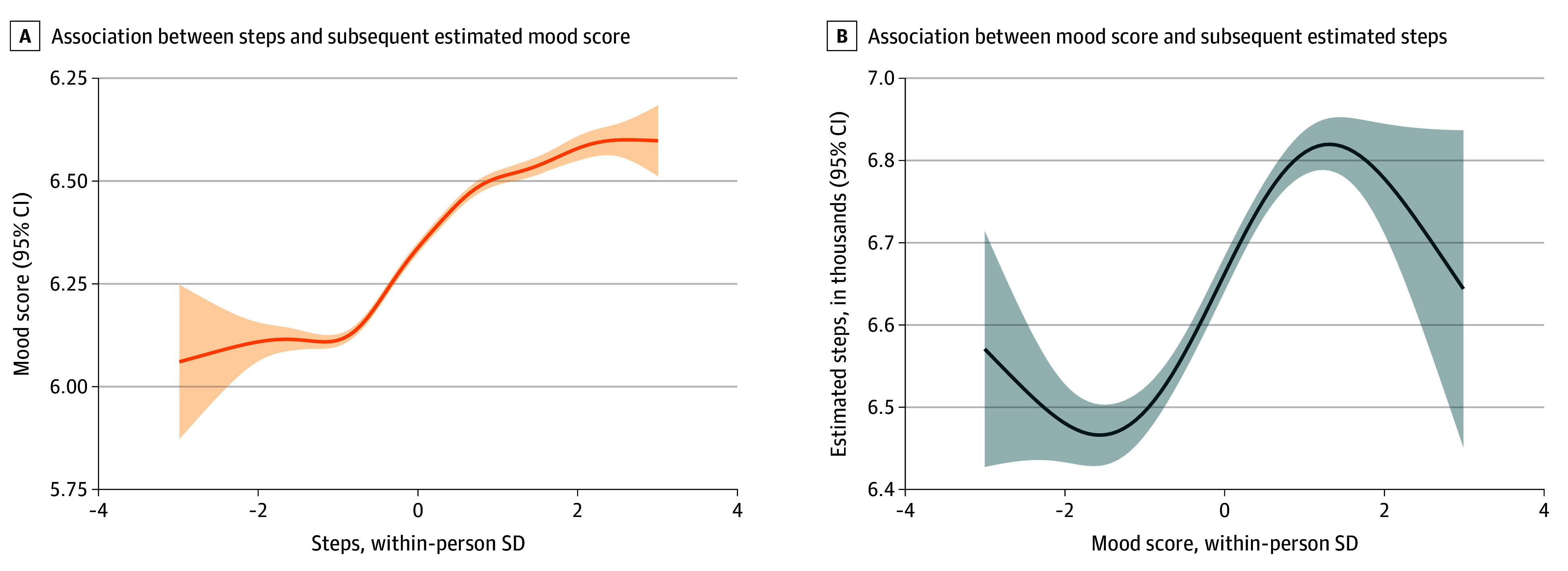
Line Graphs of Association Between Steps and Mood Score Graphs show model-estimated outcome values using generalized additive model smoothing for visualization of within-person associations. A, Association of steps time (SD from person mean; observed range, −3 to 3 SD) with subsequent estimated mood score (range, 1-10). B, Association of mood time (SD from person mean; observed range, −3 to 3 SD) with subsequent estimated steps (in thousands). Shaded regions represent 95% CIs.

We estimated the within-person association between daily steps and subsequent mood. The quadratic model provided substantially better fit than the linear model (difference in AIC = −72.38). We found a significant linear positive association between steps and mood (*b* = 0.160; 95% CI, 0.149 to 0.162; *P* < .001), along with a negative quadratic association between steps and subsequent mood (*b* = −0.022; 95% CI, −0.027 to −0.017; *P* < .001) (Table 3). These results indicate an overall positive association between steps and mood, with the magnitude of the positive association decreasing at higher than usual step counts (Figure 2A). Furthermore, we examined the association of mood with subsequent step counts, while controlling for previous-day step counts, timeline variables, and other covariates (Table 3). The quadratic model was better fit (difference in AIC = −2.93), with both the linear (*b* = 0.020; 95% CI, 0.003 to 0.030; *P* = .02) and quadratic terms (*b* = −0.010; 95% CI, −0.020 to −0.001; *P* = .03) for scaled mood being significantly associated with subsequent step counts. Overall, higher than usual mood was associated with increased step counts until approximately 1 SD above the scaled mood; however, beyond this point, the positive association of mood with subsequent steps plateaued (Figure 2B).

**Table 3.** Bidirectional Associations Between Steps-to-Mood Score

Model and variable	*b* (95% CI) [SE]	*P* value
Steps-to-mood model^a^		
Steps, scaled (linear term)	0.160 (0.149 to 0.162) [0.003]	<.001
Steps, scaled (quadratic term)	0.022 (−0.027 to −0.017) [0.003]	<.001
Mood-to-steps model^b^		
Mood, scaled (linear term)	0.020 (0.003 to 0.030) [0.008]	.02
Mood, scaled (quadratic term)	−0.010 (−0.020 to −0.001) [0.006]	.03

^a^ The steps-to-mood model was adjusted for age, sex, employment status, educational level, race, day in study, day in week, and previous day’s mood (conditional *R*^2^ = 0.457; marginal *R*^2^ = 0.181).

^b^ The mood-to-steps model was adjusted for age, sex, employment status, educational level, race, day in study, day in week, and previous day’s steps (conditional *R*^2^ = 0.437; marginal *R*^2^ = 0.099).

A combined model including both TST and step count as simultaneous factors of mood yielded effect sizes comparable with those of the separate sleep-to-mood and steps-to-mood models (eTable 3 in Supplement 1). Additionally, associations remained robust when models were stratified by baseline PHQ-9 (eTable 4 in Supplement 1) and GAD-7 severity (eTable 5 in Supplement 1).

## Discussion

In this cohort study, we used intensive, longitudinal objectively measured data to investigate the bidirectional associations between sleep and physical activity with mood among people seeking care for mental health concerns. We found an inverse U-shaped association between mood and sleep duration, where both shorter and longer than usual sleep led to lower subsequent mood. Notably, the optimal sleep range for maximizing mood varied substantially between people. Furthermore, we found a nonlinear association between mood and subsequent sleep duration. We found that increased physical activity was associated with improved mood, with the association plateauing at higher activity levels. In turn, higher than usual mood was associated with increased physical activity.

Prior studies have indicated that both short and long sleep durations are linked to poorer mental health.^9,11,38^ Our work adds to this evidence by characterizing within-person associations at daily level. We showed that, for a given individual, deviations from their own average sleep duration, whether longer or shorter, are linked to worse subsequent mood. This within-person method provides more compelling support for day-to-day behavioral-mood dynamics since it accounts for between-person variations including lifestyle and socioeconomic status and other potential confounding factors. We found that the descending part of the inverse U-shaped curve was steeper (Figure 1A), indicating that sleeping longer than usual may be more harmful than sleeping less. Additionally, the persistence of within-person sleep-mood associations across consistently short, consistently long, inconsistent, and typical sleepers suggests that daily deviation from one’s own usual sleep relates to subsequent mood regardless of overall sleep patterns. We also determined the optimal sleep duration for maximizing mood at the within-person level. Prior studies and guidelines have suggested wide and conflicting population-level optimal sleep ranges for maximizing mental health outcomes (eg, 7.5-10.5 hours or 6-8 hours).^7,11^ We showed that the optimal sleep duration for mood varies substantially from person to person, with a mean of 6.8 hours. This substantial interindividual variation in optimal sleep suggests that there may be additional value in empirically identifying person-specific targets, which may present a low-burden approach to personalizing recommendations as an adjunctive to mental health care, moving beyond the one-size-fits all approaches to improving mood through sleep. For 20.1% of participants in our study, optimal sleep durations fell outside 1 SD above or below of their observed personal mean, and this group may particularly benefit from identification of sleep targets that differ from their habitual patterns. These results also support prior studies linking higher variability in TST with worse mental health,^39,40^ indicating that consistency in sleep based on one’s own baseline may be just as important as overall sleep duration.

Additionally, we found that the structure of sleep is also significantly associated with subsequent mood. Given the same TST, multiple sleep episodes (either as daytime naps, interrupted sleep, or both) were negatively associated with subsequent mood compared with a single long consolidated episode. Multiple episodes were due to both fragmented sleep and napping behaviors. Future work should further assess how these factors may be associated with a worse mood. These findings reinforce the complex and dynamic interplay between sleep and mood. In line with work linking depressive states to hypersomnia,^41^ we found that lower than usual daily mood was associated with a longer TST the next day, following a wavelike nonlinear pattern (Figure 1B), suggesting that low mood may lead to compensatory increases in sleep.

We also found a significant positive linear association between daily step counts and subsequent mood. This positive association plateaued beyond approximately 2 SDs of the participant’s mean. Most participants remained within a moderate-step range, where increases in steps were associated with better mood. This is consistent with meta-analyses that demonstrate that even small increases in daily step count can improve depression symptoms.^42^ Prior studies were primarily between-person and seldom included step counts greater than 10 000, limiting assessment of high-activity levels.^42,43^ Our within-participant approach and inclusion of high step counts enabled us to identify a nonlinear, within-person mood benefit associated with increasing physical activity. We also found that higher than usual mood was associated with higher levels of steps the following day, with significant linear and quadratic terms, resulting in a nonlinear, wavelike pattern (Figure 2B). This indicates that mood affects behavior dynamically but nonuniformly and identifies individual differences and nonlinear trends in the link between mood and physical activity.

Sensitivity analyses indicated that the associations were consistent across varying levels of baseline depression (PHQ-9) and anxiety (GAD-7) symptoms, and that sleep and steps were independently associated with mood when modeled simultaneously. This supports previous findings that sleep and physical activity may account for unique variance in daily mood.^3^

### Limitations

Several limitations in this study should be considered. First, despite the intensive longitudinal design facilitating within-person modeling, the observational nature of the data limits us from drawing conclusions about causation; associations may be the result of shared temporal trends or underlying third variables rather than direct associations. Second, mood was measured using a once-daily self-report method, which may oversimplify complex affective states.^44^ Third, while we have adjusted for sleep fragmentation in our sleep-to-mood model through the inclusion of number of sleep episodes, we did not examine other aspects of sleep, such as sleep timing. Fourth, the mood-to-sleep and mood-to-steps models showed greater imprecision around the extreme ends of estimated associations, as reflected in wide 95% CIs. This could stem from limited sample size at the extremes of the distributions, constraining the model’s ability to detect change in the extreme ends.

## Conclusions

In this cohort study of people seeking mental health care, we used longitudinal data to uncover novel within-person associations between physical activity, sleep, and mood. We observed an inverse U-shaped association between mood and sleep duration, interindividual variations in optimal sleep duration, and negative associations between more sleep episodes and mood. We found that increasing physical activity was positively associated with mood. Furthermore, mood was associated with subsequent sleep duration and physical activity in a dynamic, nonlinear fashion. The findings highlight the importance of within-person, real-time investigations using objective measures for identifying opportunities for improving mental health. Future studies should investigate whether interventions using individualized optimum ranges identified during brief monitoring periods improve mood outcomes compared with population-level recommendations.

## Notes

Supplement 1. eMethods. Definition of Sleep Pattern Groups and Alternative Sleep Episode Type Thresholds eFigure 1. Distribution of Steps Data and Sleep Data eFigure 2. Study flow diagram describing participant inclusion from enrollment through follow-up and analyses eTable 1. Within-Person Associations of TST and Steps with Subsequent Mood, Stratified by Sleep Pattern Groups eTable 2. Sleep-Mood Model Using Alternative Nap Definition Threshold eTable 3. Combined Model: Independent Effects of TST and Steps on Subsequent Mood eTable 4. Associations of TST and Steps with Subsequent Mood, Stratified by Baseline Depression Severity (PHQ-9) eTable 5. Within-Person Associations of TST and Steps with Subsequent Mood, Stratified by Baseline Anxiety Severity (GAD-7)

Supplement 2. Data Sharing Statement
