# Supplementary material for: Sleep, Physical Activity, and Mood Among People Seeking Mental Health Care
Source: JAMA Netw Open. 2026 Mar 9;9(3):e261194. doi: 10.1001/jamanetworkopen.2026.1194 (PMC12973096; doi:10.1001/jamanetworkopen.2026.1194)
Supplement: Supplement 1. — eMethods. Definition of Sleep Pattern Groups and Alternative Sleep Episode Type Thresholds eFigure 1. Distribution of Steps Data and Sleep Data eFigure 2. Study flow diagram describing participant inclusion from enrollment through follow-up and analyses eTable 1. Within-Person Associations of TST and Steps with Subsequent Mood, Stratified by Sleep Pattern Groups eTable 2. Sleep-Mood Model Using Alternative Nap Definition Threshold eTable 3. Combined Model: Independent Effects of TST and Steps on Subsequent Mood eTable 4. Associations of TST and Steps with Subsequent Mood, Stratified by Baseline Depression Severity (PHQ-9) eTable 5. Within-Person Associations of TST and Steps with Subsequent Mood, Stratified by Baseline Anxiety Severity (GAD-7) [file jamanetwopen-e261194-s001.pdf]

Supplemental Online Content

Kulshreshtha A, Fang Y, Mills ED, Bohnert ASB, Sen S. Sleep, physical activity and mood among people seeking mental health care. *JAMA Netw Open.* 2026;9(3):e261194. doi:10.1001/jamanetworkopen.2026.1194

eMethods. Definition of Sleep Pattern Groups and Alternative Sleep Episode Type Thresholds

eFigure 1. Distribution of Steps Data and Sleep Data

eFigure 2. Study Flow Diagram Describing Participant Inclusion From Enrollment Through Follow-Up and Analyses

eTable 1. Within-Person Associations of TST and Steps with Subsequent Mood, Stratified by Sleep Pattern Groups

eTable 2. Sleep-Mood Model Using Alternative Nap Definition Threshold

eTable 3. Combined Model: Independent Effects of TST and Steps on Subsequent Mood

eTable 4. Associations of TST and Steps with Subsequent Mood, Stratified by Baseline Depression Severity (PHQ-9)

eTable 5. Within-Person Associations of TST and Steps with Subsequent Mood, Stratified by Baseline Anxiety Severity (GAD-7)

This supplemental material has been provided by the authors to give readers additional information about their work.

© 2026 Kulshreshtha A et al. *JAMA Network Open*.

eMethods. Definition of Sleep Pattern Groups and Alternative Sleep Episode Type Thresholds

Definition of Sleep Pattern Groups: We stratified participants into sleep groups based on person-level TST characteristics. We categorized participants as Consistently Poor Sleepers when their average TST was in the lowest or highest quartile (≤377 or ≥454 minutes) with low TST variability (SD < 123 minutes, below 75th percentile), indicating short or long sleep. Participants with low TST variability (SD < 123 minutes, below the 75th percentile) were further classified as Consistently Short Sleepers or Consistently Long Sleepers if their average TST fell in the lowest (≤377 minutes) or highest quartile (≥454 minutes), respectively. Inconsistent Sleepers were characterized as participants whose TST variability exceeded the 75th percentile (SD ≥ 123 minutes), indicating high sleep inconsistency. The remaining participants were labeled as Typical Sleepers. The sleep-mood association was re-estimated for each sleep group.

Alternative Sleep Episode Type Thresholds: We defined alternate thresholds to define sleep episode type and included them in our model on the effect of TST on subsequent mood to confirm the robustness of results. The thresholds were - 2 hours and 3 hours (Table 2 in manuscript). The original threshold was 3 hours (Table 2) and the alternative thresholds were -was 2 hours (eTable 1) and 3 hours (Table 2 in manuscript).

© 2026 Kulshreshtha A et al. *JAMA Network Open*.

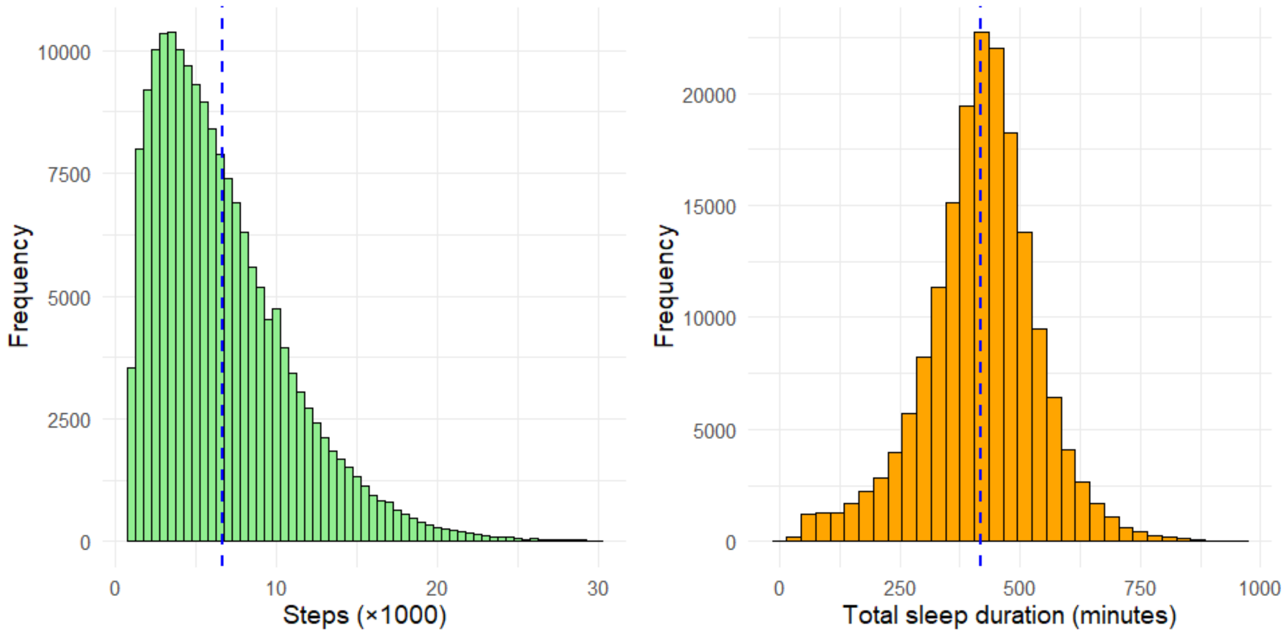

eFigure 1: Distribution of Steps Data and Sleep Duration. (a.) Distribution of steps data. The blue dotted line represents the mean steps across participants. (b.) Distribution of sleep data (in minutes). The blue dotted line represents the mean TST across participants.

© 2026 Kulshreshtha A et al. *JAMA Network Open*.

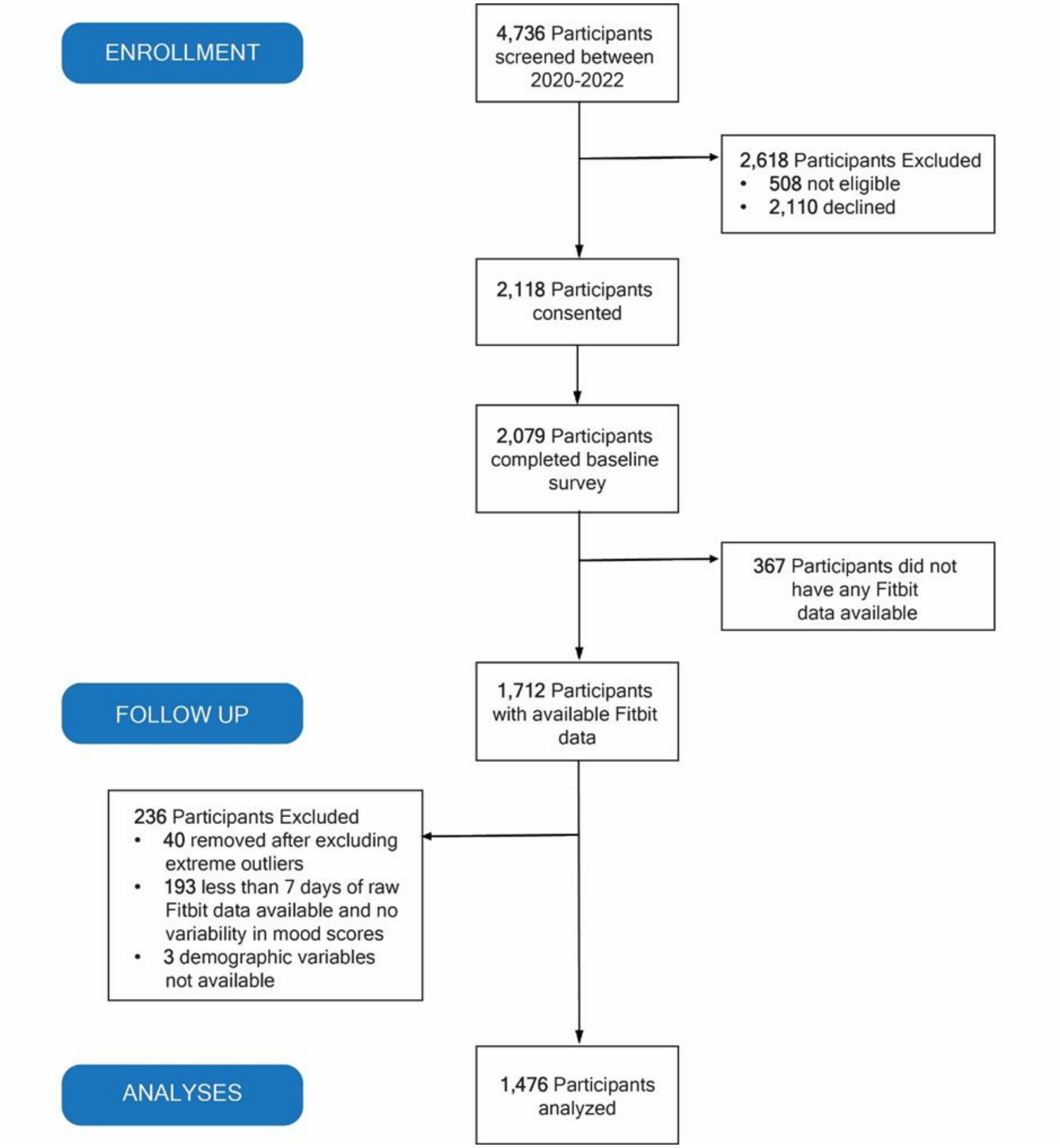

eFigure 2: Study flow diagram describing participant inclusion from enrollment through follow-up and analyses

© 2026 Kulshreshtha A et al. *JAMA Network Open*.

eTable 1: Within-Person Associations of TST and Steps with Subsequent Mood, Stratified by Sleep Pattern Groups

Predictor	Inconsistent Sleepers (N=369)			Typical Sleepers (N=541)			Consistently Short Sleepers (N=249)			Consistently Long Sleepers (N=292)		
	b (95% CI)	SE	P value	b (95% CI)	SE	P value	b (95% CI)	SE	P value	b (95% CI)	SE	P value
**TST, scaled (linear)**	-0.03 (-0.05, -0.02)	.008	<.001	0.006 (-0.003, 0.02)	0.008	.21	0.02 (0.008, 0.04)	0.008	.003	-0.08 (-0.02, 0.005)	0.007	.25
**TST, scaled (quadratic)**	-0.03 (-0.04, -0.02)	0.005	<.001	-0.02 (-0.03, -0.01)	0.003	<.001	-0.03 (-0.05, -0.02)	0.006	<.001	-0.03 (-0.04, -0.02)	0.005	<.001
**Sleep Episode Type (non-nap)**	-0.13 (-0.18, -0.07)	0.03	<.001	-0.12 (-0.16, -0.08)	0.02	<.001	-0.06 (-0.12, -0.003)	0.03	.04	-0.22 (-0.28, -0.16)	0.03	<.001
**Sleep Episode Type (nap)**	-0.16 (-0.2, -0.12)	0.02	<.001	-0.18 (-0.21, -0.15)	0.02	<.001	-0.17 (-0.22, -0.12)	0.03	<.001	-0.2 (-0.24, -0.16)	0.02	<.001
**Sleep Episode Type (both)**	-0.25 (-0.34, -0.17)	0.05	<.001	-0.3 (-0.39, -0.21)	0.05	<.001	-0.12 (-0.27, 0.03)	0.08	.11	-0.32 (-0.44, -0.2)	0.06	<.001
Models adjusted for age, sex, employment status, educational level, race, day in study, day in week, previous day's mood.												

© 2026 Kulshreshtha A et al. *JAMA Network Open*.

eTable 2: Sleep-Mood Model Using Alternative Nap Definition Threshold. Sleep episode threshold (2 hr)

Model	Outcome	Predictor	b (95% CI)	SE	P value
**Sleep → Mood[a]**	Subsequent mood	Total sleep time, scaled (linear term)	−0.002 (-0.008, 0.004)	0.003	.52
		Total sleep time, scaled (quadratic term)	−0.03 (-0.03, -0.02)	0.002	<.001
		Sleep Episode Type (non-nap)	−0.12 (-0.15, -0.09)	0.02	<.001
		Sleep Episode Type (nap)	−0.18 (-0.2, -0.16)	0.01	<.001
		Sleep Episode Type (both)	−0.24 (-0.3, -0.18)	0.03	<.001

[a] Model adjusted for age, sex, employment status, educational level, race, day in study, day in week, previous day's mood.

© 2026 Kulshreshtha A et al. *JAMA Network Open*.

eTable 3: Combined Model: Independent Effects of TST and Steps on Subsequent Mood

Model	Predictor	b (95% CI)	SE	P value
**Sleep, Steps → Mood[a]**	Total sleep time, scaled (linear term)	0.02 (0.008, 0.02)	0.003	<.001
	Total sleep time, scaled (quadratic term)	−0.02 (-0.02, -0.15)	0.002	<.001
	Sleep Episode Type (non-nap)	−0.1 (-0.13, -0.08)	0.01	<.001
	Sleep Episode Type (nap)	−0.15 (-0.17, -0.13)	0.01	<.001
	Sleep Episode Type (both)	−0.21 (-0.26, -0.16)	0.03	<.001
	Steps, scaled (linear term)	0.15 (0.14, 0.15)	0.003	<.001
	Steps, scaled (quadratic term)	-0.02 (-0.03, -0.02)	0.003	<.001

[a] Model adjusted for age, sex, employment status, educational level, race, day in study, day in week, previous day's mood.

© 2026 Kulshreshtha A et al. *JAMA Network Open*.

eTable 4: Associations of TST and Steps with Subsequent Mood, Stratified by Baseline Depression Severity (PHQ-9)

Model	Predictor	PHQ-9 <10 (N=499)			PHQ-9 ≥10 (N=977)		
		b (95% CI)	SE	P value	b (95% CI)	SE	P value
**Sleep → mood[a]**	TST, scaled (linear term)	0.004 (-0.005, 0.01)	0.005	.38	-0.005 (-0.01, 0.003)	0.004	.25
	TST, scaled (quadratic term)	-0.03 (-0.03, -0.02)	0.004	<.001	-0.03 (-0.03, -0.02)	0.003	<.001
	Sleep Episode Type (non-nap)	-0.12 (-0.16, -0.08)	0.02	<.001	-0.14 (-0.17, -0.1)	0.02	<.001
	Sleep Episode Type (nap)	-0.13 (-0.16, -0.1)	0.02	<.001	-0.2 (-0.23, -0.18)	0.01	<.001
	Sleep Episode Type (both)	-0.21 (-0.29, -0.13)	0.04	<.001	-0.3 (-0.37, -0.24)	0.03	<.001
**Steps → mood[a]**	Steps, scaled (linear term)	0.14 (0.13, 0.15)	0.005	<.001	0.17 (0.16, 0.18)	0.004	<.001
	Steps, scaled (quadratic term)	-0.02 (-0.03, -0.02)	0.004	<.001	-0.02 (-0.03, -0.02)	0.003	<.001

[a] Models adjusted for age, sex, employment status, educational level, race, day in study, day in week, previous day's mood.

© 2026 Kulshreshtha A et al. *JAMA Network Open*.

eTable 5: Within-Person Associations of TST and Steps with Subsequent Mood, Stratified by Baseline Anxiety Severity (GAD-7)

Model	Predictor	GAD-7 <10 (N=587)			GAD-7 ≥10 (N=889)		
		b (95% CI)	SE	P value	b (95% CI)	SE	P value
**Sleep → mood**[a]	TST, scaled (linear term)	-0.006 (-0.02, 0.003)	0.005	.18	0.001 (-0.008, 0.01)	0.005	.78
	TST, scaled (quadratic term)	-0.03 (-0.04, -0.02)	0.003	<.001	-0.03 (-0.03, -0.02)	0.003	<.001
	Sleep Episode Type (non-nap)	-0.13 (-0.17, -0.1)	0.02	<.001	-0.13 (-0.16, -0.09)	0.02	<.001
	Sleep Episode Type (nap)	-0.15 (-0.18, -0.13)	0.01	<.001	-0.2 (-0.23, -0.17)	0.01	<.001
	Sleep Episode Type (both)	-0.26 (-0.33, -0.2)	0.04	<.001	-0.27 (-0.34, -0.2)	0.04	<.001
**Steps → mood**[a]	Steps, scaled (linear term)	0.14 (0.13, 0.15)	0.005	<.001	0.17 (0.16, 0.17)	0.005	<.001
	Steps, scaled (quadratic term)	-0.03 (-0.04, -0.02)	0.004	<.001	-0.02 (-0.03, -0.01)	0.004	<.001

[a] Models adjusted for age, sex, employment status, educational level, race, day in study, day in week, previous day's mood.

© 2026 Kulshreshtha A et al. *JAMA Network Open*.
