# Supplementary material for: Sleep, Physical Activity, and Mood Among People Seeking Mental Health Care
Source: JAMA Netw Open. 2026 Mar 9;9(3):e261194. doi: 10.1001/jamanetworkopen.2026.1194 (PMC12973096; doi:10.1001/jamanetworkopen.2026.1194)
Supplement: Supplement 2. — Data Sharing Statement [file jamanetwopen-e261194-s002.pdf]

# Data Sharing Statement

Kulshreshtha. Sleep, Physical Activity, and Mood Among People Seeking Mental Health Care. *JAMA Netw Open*. Published March 09, 2026. doi:10.1001/jamanetworkopen.2026.1194

**Data**
**Data available:** Yes
**Data types:** Deidentified participant data
**How to access data:** Data may be requested and accessed through the precision health analytics platform, at the following website: https://sites.lsa.umich.edu/aidhi-data-details/data-access-2/
**When available:** With publication

**Supporting Documents**
**Document types:** Statistical/analytic code
**How to access documents:** Specific requests for code can be sent to Aishani Kulshreshtha (aishanik@umich.edu)
**When available:** With publication

**Additional Information**
**Who can access the data:** Individuals may access the data through the Precision Health Analytics Platform, which does require specific criteria to be met in order to ensure data safety and patient privacy. Requirements can be viewed at this site: https://sites.lsa.umich.edu/aidhi-data-details/data-access-2/
**Types of analyses:** Once the above criteria are met, investigators are able to analyze data according to the pre-specified purpose.
**Mechanisms of data availability:** Data will be made available when specified criteria are met (https://sites.lsa.umich.edu/aidhi-data-details/data-access-2/)
